# New Guideline-Directed Treatments for Heart Failure: Navigating Through the Multiple Turns of Everyday Clinical Practice

**DOI:** 10.1016/j.jaccas.2021.11.006

**Published:** 2022-01-05

**Authors:** Luca Paolucci, Francesco Grigioni, Valeria Cammalleri, Gian Paolo Ussia, Maurice Enriquez-Sarano

**Affiliations:** a Units of Cardiovascular Science, Department of Medicine, University Campus Bio-Medico of Rome, Rome, Italy; b Valve Science Center, Minneapolis Heart Institute, Minneapolis, Minnesota, USA

**Keywords:** guidelines, heart failure, mitral regurgitation, sodium-glucose cotransporter 2 inhibitors, transcatheter edge-to-edge mitral valve repair, tricuspid regurgitation

## Abstract

The new European guidelines for the treatment and management of heart failure (HF) introduce several new recommendations. The revised HF definition has abolished the term “mid-range,” introducing the new concept of “mildly reduced” ejection fraction (EF), which now deserves consideration for therapies previously confined to reduced EF (HF with reduced EF). Following the introduction of sodium-glucose cotransporter 2 inhibitors, physicians should now combine up to 4 different drugs to improve HF with reduced EF prognosis, leading to new issues regarding tolerance and adherence to therapy. Transcatheter treatments of mitral and tricuspid regurgitation are progressively gaining increasing consideration among nonpharmacological strategies. Dedicated therapies for HF with preserved EF are still lacking. These are only some of the most relevant changes provided by European guidelines on HF that are addressed in the present editorial, taking into account the most updated American recommendations.

## Corresponding Author

**Figure:**
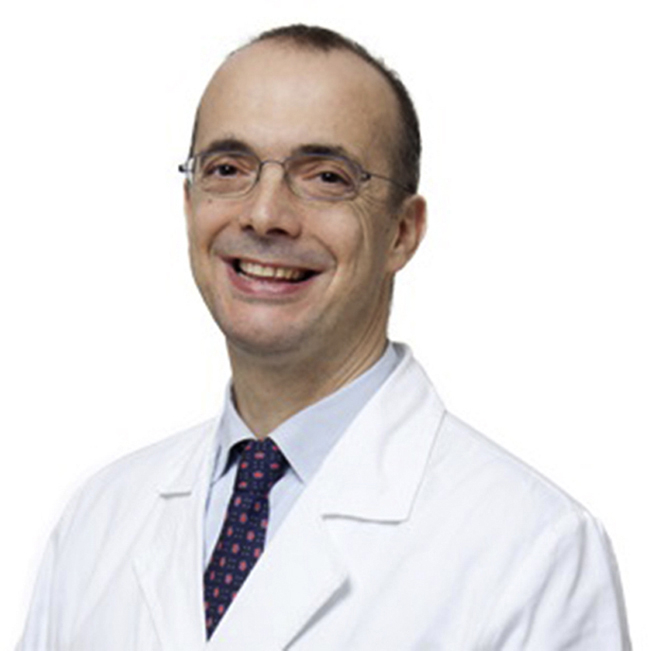

The recently published guidelines from the European Society of Cardiology (ESC) for the diagnosis and treatment of heart failure (HF) introduced several, but relevant, changes, mostly coming from prospective randomized trials.^1^ At the same time, the increasing number of recommendations implied a parallel increase in the difficulty of adapting everyday clinical practice to guidelines.

In the present editorial, we aim at summarizing most of the new key recommendations as well as providing practical pieces of advice on how to incorporate them in our daily patient management.

## Redefining patients with HF: Therapeutic implications

Until now, we categorized HF patients according to left ventricular ejection fraction (LVEF): reduced (heart failure with reduced ejection fraction [HFrEF]: <40%), mid-range (HF with mildly reduced EF: 40%-49%), and preserved (heart failure with preserved ejection fraction [HFpEF]: >50%). The rationale was based on the therapeutic benefits available at the time only for LVEF <40%. In the new ESC guidelines, this definition evolved, highlighting that therapies beneficial in HFrEF can also be effective in patients with LVEF ranging between 40% and 50%.^1^ From now on, we should therefore change our perception of HF patients with LVEF from 40% to 50%, replace the term “mid-range” with “mildly reduced” LVEF (HF with mildly reduced EF), and offer them HFrEF therapies.

No treatment has convincingly been shown to improve the prognosis of HFpEF because it includes a more heterogeneous group of patients. For instance, HFpEF with supernormal (>65%) LVEF may often be due to amyloidosis or hypertrophic cardiomyopathy (which can take advantage of specific therapies). Hopefully, specifically designed randomized studies will lead, in the future, to successfully characterize and approach (currently) untreatable phenotypes of HFpEF.

## The new paradigm(s) in the treatment of HFrEF

In HFrEF, the new sodium-glucose cotransporter 2 (SGLT2) inhibitors dapaglifozin and empaglifozin earned a central role. Despite there being no definitive consensus about how these molecules can provide cardiac benefit, both improve HF outcome, regardless of the presence of diabetes.^1^ A recent trial suggests these molecules may be effective even in patients with an LVEF >40%.^2^

Because we had already 4 different classes of drugs with a Class I recommendation in HFrEF (besides SGLT2 inhibitors), the issue is now how to manage an increasingly complex armamentarium. The task becomes even more challenging when we need to add on top of disease-modifying drugs symptomatic therapies, such as diuretics. A recent consensus from the American College of Cardiology gives us directions.^3^ All patients with a new diagnosis of symptomatic HFrEF should receive a beta-blocker and concomitantly an angiotensin-converting enzyme (ACE) inhibitor, angiotensin receptor blocker, or angiotensin receptor-neprilysin inhibitor (ARNI). Each one of these inhibitors can be first-line therapy initiated as soon as possible and should be up-titrated every 2 weeks to the target dose. Patients shifting from ACE inhibitors to ARNIs should receive a starting dose according to the previous ACE inhibitor dose (high dose: 46/51 mg; low/medium dose: 24/26 mg). Notably, in ACE inhibitor-naïve patients, a “run-in” phase is no longer mandatory, and a direct-to-ARNI approach can be adopted. Choosing this strategy, the most common adverse effects are related to hypotension, while angioedema is infrequent.

In addition to ARNIs or ACE inhibitors and beta-blockers, ESC guidelines state that both aldosterone antagonists and SGLT2 inhibitors should be routinely used in all HFrEF patients.^1^ Despite generally being well tolerated, SGLT2 inhibitors should be administered cautiously in patients with symptomatic hypotension, or treated with other antidiabetics or diuretics. Moreover, the prescription of these molecules should follow a careful evaluation of the estimated glomerular filtration rate and potassium plasma levels.^3^

In patients with sinus rhythm and heart rate >70 beats/min, ivabradine may represent a valuable option if a target beta-blockers dosage is achieved or not tolerated.^1^^,^^3^

Hydralazine/isosorbide dinitrate is recommended in self-identified African American patients who are still symptomatic despite optimal medical therapy.^1^^,^^3^

**Figure 1:**
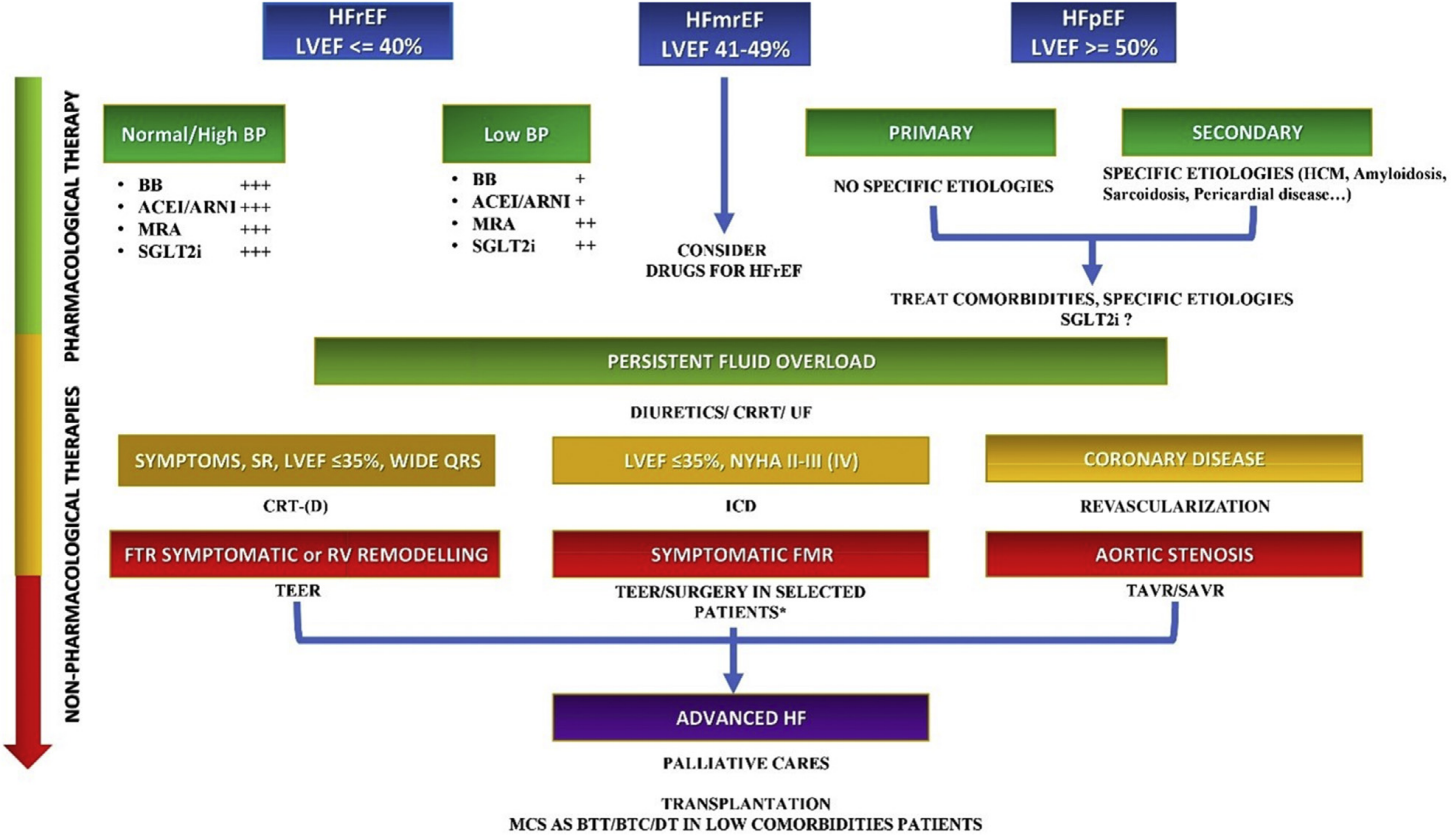
Proposal of a Therapeutic Algorithm in HF According to Clinical and Instrumental Characteristics This proposal is based on the most recent European guidelines and American recommendations, taking into account the authors’ clinical experience. The **asterisk** indicates moderate left ventricular dysfunction and unfavorable anatomy for transcatheter edge-to-edge repair (TEER). **+++** indicates safe; **++** indicates relatively safe, and **+** indicates use with caution. ACEI = angiotensin-converting enzyme inhibitors; ARNI = angiotensin receptor-neprilysin inhibitors; BB = beta-blockers; BP = blood pressure; BTC = bridge to candidacy; BTT = bridge to transplantation; CRRT = continuous renal replacement therapy; CRT-(D) = cardiac resynchronization therapy with defibrillator; DT = destination therapy; FMR = functional mitral regurgitation; FTR = functional tricuspid regurgitation; HCM = hypertrophic cardiomyopathy; HF = heart failure; HFmrEF = heart failure with mildly reduced ejection fraction; HFpEF = heart failure with preserved ejection fraction; HFrEF = heart failure with reduced ejection fraction; ICD = implantable cardioverter-defibrillator; LVEF = left ventricular ejection fraction; MCS = mechanical circulatory support; MRA = mineralocorticoid receptor antagonists; NYHA = New York Heart Association classification; RV = right ventricular; SAVR = surgical aortic valve replacement; SGLT2i = sodium-glucose co-transporter 2 inhibitors; SR = sinus rhythm; TAVR = transcatheter aortic valve replacement; UF = ultrafiltration.

Focusing on the specific patients’ clinical profiles can help plan our strategy.^4^ A patient with low blood pressure and heart rate may particularly benefit from high dosages of SGLT2 inhibitors and mineralocorticoid receptor antagonists (which have a poor effect on systemic blood pressure and no effects on heart rate), while beta-blockers and ACE inhibitors or ARNIs should be introduced more carefully. Patients with severe chronic kidney disease require specific attention because although they grossly represent 25% of the HF population, they are generally excluded from clinical trials. Obtaining a full guideline-directed medical therapy in patients with an estimated glomerular filtration rate <20 to 30 mL/min/1.73 m^2^ may be challenging, especially if hyperkalemia coexists. While mineralocorticoid antagonists should be carefully administered in these patients, beta-blockers are relatively safe.^4^ Most updated recommendations for HF treatment have been briefly summarized in Figure 1.

Other drugs can be chosen in other specific clinical settings (eg, ferric carboxymaltose in patients suffering concomitant iron deficiency),^1^ and others will be probably available shortly.

Once we finally adopt a pharmacological strategy, we need to ascertain medical adherence, which is multifactorial and involves several aspects besides compliance. Indeed, the complexity of the therapy, the coexistence of comorbidities, and the occurrence of side effects are not the only determinants of medical adherence. Other barriers include socioeconomic status, the lack of social assistance, and the reduced efficiency of health care programs. Medical adherence needs specific political and social interventions, especially in the setting of a chronic disease such as HFrEF. The American recommendations provide specific indications to improve medication adherence, including simplified therapeutic regimens, lower-cost medications, dedicated technological tools (as smartphones), and periodical monitoring of patients’ compliance.^3^

## Behind pharmacological treatments

Implantable cardioverter-defibrillators (in LVEF <35% following 3 months of optimized therapy) and cardiac resynchronization therapy (in symptomatic patients with LVEF <35% and wide [130/150 ms] QRS complex, especially with left bundle branch block morphology) remain a cornerstone for HFrEF treatments.

Transcatheter edge-to-edge mitral valve repair and surgical or percutaneous aortic valve replacement are now part of dedicated pathways in patients with HFrEF and valvular disease. Filling the criteria of the COAPT (Cardiovascular Outcomes Assessment of the MitraClip Percutaneous Therapy for Heart Failure Patients With Functional Mitral Regurgitation) trial (LVEF 20%-50%, LV end-systolic diameter <70 mm, systolic pulmonary pressure <70 mm Hg, absence of significant right ventricular dysfunction or hemodynamic instability) is currently recommended to increase the probability of procedural success, while more recent data focus on avoiding residual mitral regurgitation.^5^

Transcatheter treatment of functional tricuspid regurgitation opened a new and promising field in HFrEF and HFpEF. Patients’ selection and optimal timing for intervention remain debated and guidelines recommend being cautious, while the scientific community is welcoming this new opportunity.

## What’s next? The challenge of “optimal treatment”

Because of the availability of so many different drugs with a Class I recommendation, not all patients will be exposed to all of them. The consequences of drugs under treatment or withdrawal are unknown, and no specific strategy aiming at introducing in a given patient the full medical armamentarium has been demonstrated to be preferable.

Any new therapy for HF must be compared with the “current optimal standard of care.” The definition of “optimal treatment” and “adherence” will play a central role in defining the quality of future trials.

It is likely that guideline-directed but patient-tailored treatments will become the next step to follow for all of us, aiming at identifying what is “optimal” in every specific patient at a given point in time. At least we hope.

## Funding Support and Author Disclosures

The authors have reported that they have no relationships relevant to the contents of this paper to disclose.

## References

[1] McDonagh T.A., Metra M., Adamo M. (2021). 2021 ESC guidelines for the diagnosis and treatment of acute and chronic heart failure: developed by the Task Force for the diagnosis and treatment of acute and chronic heart failure of the European Society of Cardiology (ESC) with the special contribution of the Heart Failure Association (HFA) of the ESC. Eur Heart J.

[2] Anker S.D., Butler J., Filippatos G. (2021). Empagliflozin in heart failure with a preserved ejection fraction. N Engl J Med.

[3] Maddox T.M., Januzzi J.L.J., Allen L.A. (2021). 2021 Update to the 2017 ACC expert consensus decision pathway for optimization of heart failure treatment : answers to 10 pivotal issues about heart failure with reduced ejection fraction. J Am Coll Cardiol.

[4] Rosano G.M.C., Moura B., Metra M. (2021). Patient profiling in heart failure for tailoring medical therapy. A consensus document of the Heart Failure Association of the European Society of Cardiology. Eur J Heart Fail.

[5] Kar S., Mack M.J., Lindenfeld J. (2021). Relationship between residual mitral regurgitation and clinical and quality-of-life outcomes after transcatheter and medical treatments in heart failure. Circulation.

